# Joint Analysis of Multiple Metagenomic Samples

**DOI:** 10.1371/journal.pcbi.1002373

**Published:** 2012-02-16

**Authors:** Yael Baran, Eran Halperin

**Affiliations:** 1 School of Computer Science, Tel-Aviv University, Tel-Aviv, Israel; 2 School of Computer Science and the Department of Molecular Microbiology and Biotechnology, Tel-Aviv University, Tel-Aviv, Israel; 3 International Computer Science Institute, Berkeley, California, Unites States of America; University of Oxford, United Kingdom

## Abstract

The availability of metagenomic sequencing data, generated by sequencing DNA pooled from multiple microbes living jointly, has increased sharply in the last few years with developments in sequencing technology. Characterizing the contents of metagenomic samples is a challenging task, which has been extensively attempted by both supervised and unsupervised techniques, each with its own limitations. Common to practically all the methods is the processing of single samples only; when multiple samples are sequenced, each is analyzed separately and the results are combined. In this paper we propose to perform a combined analysis of a set of samples in order to obtain a better characterization of each of the samples, and provide two applications of this principle. First, we use an unsupervised probabilistic mixture model to infer hidden components shared across metagenomic samples. We incorporate the model in a novel framework for studying association of microbial sequence elements with phenotypes, analogous to the genome-wide association studies performed on human genomes: We demonstrate that stratification may result in false discoveries of such associations, and that the components inferred by the model can be used to correct for this stratification. Second, we propose a novel read clustering (also termed “binning”) algorithm which operates on multiple samples simultaneously, leveraging on the assumption that the different samples contain the same microbial species, possibly in different proportions. We show that integrating information across multiple samples yields more precise binning on each of the samples. Moreover, for both applications we demonstrate that given a fixed depth of coverage, the average per-sample performance generally increases with the number of sequenced samples as long as the per-sample coverage is high enough.

## Author Summary

Microorganisms are extremely abundant and diverse, and occupy almost every habitat on earth. Most of these habitats contain a complex mixture of many different microorganisms, and the characterization of these metagenomic mixtures, in terms of both taxonomy and function, is of great interest to science and medicine. Current sequencing technologies produce large numbers of short DNA reads copied from the genomes of a metagenomic sample, which can be used to obtain a high resolution characterization of such samples. However, the analysis of such data is complicated by the fact that one cannot tell which sequencing reads originated from the same genome. We show that the joint analysis of multiple metagenomic samples, which takes advantage of the fact that the samples share common microbial types, achieves better single-sample characterization compared to the current analysis methods that operate on single samples only. We demonstrate how this approach can be used to infer microbial components without the use of external sequence data, and to cluster sequencing reads according to their species of origin. In both cases we show that the joint analysis enhances the average single-sample performance, thus providing better sample characterization.

## Introduction

Metagenomic samples are pooled samples of the genomes of multiple microorganisms living in the same environment. They can be taken either from the outer environment or from microbial populations colonizing other living organisms. Metagenomic studies focus on the taxonomic and functional characterization of the microbial populations contained in such samples. These studies have been boosted by advances in Next Generation Sequencing (NGS) technologies. Particularly, Whole Genome Shotgun (WGS) sequencing provides reads sampled randomly along the genomes, and enables simultaneous phylogenetic and functional analysis of the samples. Although WGS datasets contain plenty of information, they are hard to decipher, as we will further explain below. In a nutshell, the natural way to explore their composition is by aligning the sequencing reads against known databases of whole genomes or of marker genes, however these databases are seriously limited and biased. In addition, one cannot a-priori tell which reads originated from the same genome, and therefore many methods attempt to cluster the reads according to species of origin as a preliminary stage; unsupervised binning methods face an especially hard challenge, and are currently practiced mostly on extremely simple or simulated datasets.

Along with the increasing availability of single-metagenome WGS datasets, datasets consisting of multiple metagenomic samples are also becoming abundant. These datasets typically include samples taken from similar environments, such as ocean water sampled from different locations or depths [1], or microbiomic samples taken from a group of human individuals [2]. To date, the primary analysis of the resulting sequences is performed separately for each sample. Our principal observation is that combining information from multiple samples improves the characterization of each of the samples. We give two demonstrations of this principle: First, we present a method for the unsupervised characterization and quantification of shared hidden components across samples. Second, we present a binning method that operates on multiple samples simultaneously in order to achieve high per-sample precision.

We consider an unsupervised learning approach, in which we aim at learning the shared components of the different samples in an attempt to answer the prominent question of metagenomics, “what's in the mix”, without relying on any prior knowledge. While the use of stored sequences of whole genomes [3] or of marker genes, such as the 16S rRNA subunit [4], is currently the most effective way of analyzing large-scale metagenomic samples, it is considerably hindered by the incompleteness of existing databases: In addition to including only a small fraction of the species expected to be found in the samples, the set of species which these databases do include is highly biased, and this bias in turn causes a bias in the analysis results. Supervised analyses also often assume that the properties of the samples which are of biological or medical interest correspond to known taxonomic or functional annotations, although this is not necessarily the case. An intriguing counter-example is the recently discovered *enterotype* classes [5], which are three robust classes to which human gut metagenomic samples can be classified. Although generated using a supervised technique, these classes are characterized by a complex combination of the abundance of many bacterial species, which do not correspond to specific taxonomic units.

Aiming to avoid these disadvantages, we developed a method for the inference of hidden components within the data, which leverages on the fact that these unknown components are shared by the different samples. Each of the components is characterized by its sequence composition pattern, specifically the frequency of different short 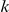-mer words in the sequence, which is known to characterize bacteria at different phylogenetic scales [6], [7]. Due to the unsupervised nature of the method, we do not expect the components to represent any easily-interpretable biological entity, but instead to provide a composite characterization of the samples. Unlike the enterotypes clustering procedure, our method does not require an alignment stage and does not classify the samples to distinct classes. Instead, we search for the best components that explain the data, and each sample is assigned a distribution over these components; this is done by utilizing Probabilistic Latent Semantic Analysis (PLSA) [8], a technique applied to fields such as information retrieval and natural language processing. Despite these differences, there are some correlations between the inferred components and the enterotypes, which we mention in the Discussion.

Unsupervised component estimation can be used for multiple purposes, and we choose to demonstrate its applicability to a new paradigm for studying statistical association between metagenomic content and phenotypes, which we now introduce. We look for DNA words - long 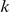-mers - whose abundance in the sequencing reads of the different samples correlate with the phenotype at question. For large enough 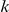, differences in the abundance of certain 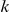-mers would capture differences in the abundance of specific species, genes or functional domains which cause the phenotype or are affected by it.

The proposed framework is analogous to the widely used paradigm of Genome Wide Association Studies (GWAS), which is used to test for associations between genetic variants in the human genome and phenotypes. In a typical GWAS the frequency of millions of variants, spanning the entire genome, is compared between a group of cases and a group of controls, and variants whose frequencies differ significantly between the two are considered to be statistically associated with the condition. In the context of metagenomic association, the 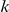-mers are analogous to the genetic variants studied in GWAS, and in both applications the goal is to find statistically significant associations between the measured variants and the condition. However, while GWAS searches for specific mutations which are associated with increased risk for the condition, we aim to capture modifications in the bacterial composition - functional or taxonomic - which are associated with the disease. As in the case of GWAS, the advantages of our approach are its computational efficiency, statistical rigor, cross-study comparability, and the fact that it does not require a supervised stage or comparison to existing databases.

Interestingly, when testing this approach on a publicly available dataset [2] containing 124 deeply sequenced samples of human gut microbiomes collected as part of the MetaHIT (Metagenomics of the Human Intestinal Tract) project, we found that the abundance of a large fraction of the 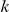-mers vary with some of the phenotypes, even for 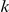 as small as 3. In the GWAS context this is known as a case of stratification: the null hypothesis of equal distribution between phenotype groups does not hold for the typical variant. For example, when the case and control groups have different ethnic composition, the minor allele frequency of an exceptionally large number of Single Nucleotide Polymorphisms (SNPs) may appear correlated with the disease, but these correlations reflect the fundamental genetic difference between the groups, instead of being relevant to the disease.

In order to correct for the stratification and conduct a proper association analysis, we integrate into the association test the estimates provided by the probabilistic model, specifically the estimated proportion of each component within each sample. We chose to characterize the components according to the short 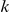-mers distribution in the samples. Recently, Meinicke et al. [9] propose to model the 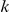-mers distribution of a single metagenomic sample as a mixture over the distributions of already-sequenced genomes; however, the use of multiple samples in our method allows our method to remain unsupervised.

As a second demonstration of the joint analysis approach we consider the task of binning sequence reads into an unknown set of species. Binning is an important preliminary step for further metagenomic analysis, and has been heavily investigated in the past few years, including the development of multiple unsupervised methods [10]–[16]; however, all existing methods operate on single samples only. We suggest an unsupervised coverage-based approach, and demonstrate that when the samples share a common species core, information can be integrated between them to improve binning precision. In other words, if one wishes to bin a given sample, then the simultaneous binning of other samples would yield better precision for the original sample. Moreover, we show that for a fixed depth of coverage, dividing the sequencing reads between additional related samples improves precision on the sample of interest.

## Methods

### Association test for metagenomes

Over the last few years, there have been many reports of associations between the content of metagenomic samples, especially human microbiomic samples, and phenotypes. Different studies report associations with different properties of the samples, such as the abundance of certain taxonomic units, mostly phyla and species, the overall taxonomic and functional diversity of the samples, and the abundance of certain genes or groups of genes, such as those participating in specific metabolic pathways (see Turnbaugh et al. [17] for a comprehensive study of obesity which tested most of these properties). In addition, dimensionality reduction techniques such as PCA [18] are often used on top of the raw data. While examining many properties of the samples allows to capture a wide range of associations, it is not always possible to accumulate results over different studies, in order to perform meta-analysis. In addition, it is hard to perform a rigorous statistical analysis, and especially to control for multiple hypotheses, when many different types of tests are carried out.

As a more rigorous approach, we propose to test the abundance of all possible DNA words of a fixed length for association with the phenotype. This test examines a limited but well-defined group of variants, and hence while it is not expected to capture the entire spectrum of possible associations, its results are statistically robust and easy to compare across studies and accumulate for future meta-analysis studies. It does not require alignment or comparison against any existing database, and therefore it can capture associations with unannotated sequences; due to the latter, the test is also fast and easy to implement.

Formally, for a given value of 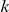, the number of occurrences of all 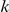-mers across each of the samples are normalized to obtain sample-specific relative abundances. The counts of complementary 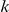-mers are summed together as they are indistinguishable in the sequencing data. We denote by 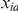 the relative abundance of 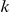-mer 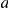 in sample 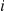, and by 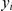 the phenotype of sample 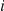. We test the association between the 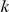-mer and the phenotype by fitting a regression model of the form
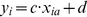
(1)For a given phenotype 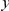 we solve the model for each 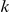-mer 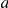 by generating the appropriate vector 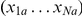, where 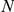 is the number of samples. We use simple regression and logistic regression for continuous and dichotomous phenotypes respectively.

In the Results section we report that some phenotypes are correlated with a large fraction of the 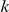-mers. These correlations reflect large-scale differences in the genetic composition of the samples between the phenotype groups; specifically, a plausible assumption is that there exists a group of common microbial components, and that each sample is a mixture of these components, in unique proportions. The components might be, for example, different bacterial phyla, and a certain phenotype might correlate with a higher proportion of a certain phylum; since there are differences in sequence composition between the phyla, this would cause phenotype-correlated differences in the distributions of many 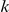-mers. However, we are interested not in the large-scale variation, but in the 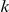-mers which remain correlated with the phenotype after taking this variation into account. Assuming there are 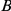 components and denoting by 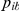 the proportion of component 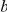 in sample 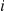, 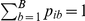, the estimation of the matrix 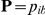 would allow us to construct a corrected model:
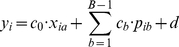
(2)


This equation is similar to equation 1 but includes the additional confounding components as covariates. For a given phenotype 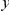, we again solve the model for each k-mer while keeping the covariate expressions fixed. Due to this addition, the association of a 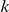-mer whose association with the phenotype is explained by the covariates will not be statistically significant, as desired. Note that 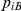 is not included in the equation because of the linear dependency 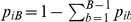.

### Probabilistic model for stratification

In order to estimate 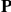 we use the following probabilistic model. We assume that the sequencing reads for 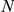 metagenomic samples are given, and that the DNA content of the samples is composed of a common set of components; each read has originated from one of the components, and each component is characterized by a typical distribution over the group of all possible 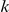-mers in the sequence, for some fixed small value of 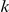 (e.g., 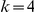). The model is parametrized by two row-stochastic matrices, 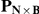 and 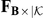: the 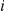
th row of 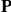, denoted 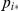, defines a sample-specific multinomial distribution over all components, and the 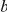
th row of 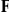, denoted 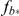, defines a component-specific multinomial distribution over 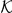, the group of all 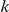-mers. When we sample a short 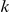-mer from a random position on the reads of sample 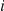, we first sample a component 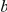 according to the distribution 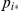, and then sample the 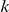-mer according to the distribution 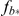. Being defined as general multinomial distribution, some entries in 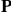 and 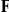 may have a zero value; in particular, some components might not be represented in some of the samples.

We note that while the previous subsection discussed long 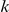-mers (e.g., 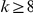), which are each tested for association with the phenotypes, in this section we use short 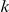-mers as characteristics of the components we attempt to learn. Specifically, we chose to use 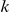 = 4 since it has been shown that 4-mer distributions are characteristic of phylogenetic units [6], [7], and since the 4-mer distribution captures both the codon distribution and possible codon biases.

We now turn to calculate the likelihood of the metagenomic data. Since the model explains the 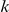-mer distribution in the reads, we extract the first 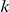-mer from each read, and denote by 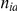 the number of times 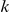-mer 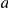 was extracted from sample 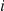. The likelihood of the counts data *R* is
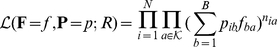
(3)where 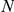 is the number of samples, 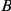 is the number of components, and 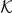 is the group of all possible 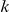-mers, 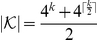 as the counts for complementary 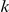-mers are joined. Our goal is to estimate the distributions in the matrix 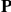, which are the distributions over the components for each sample.

We note that there is a simple relation between 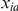 and the above notation, given by 
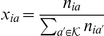
. Furthermore, under the model assumptions, we have that 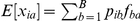. One can verify that if there is a solution such that 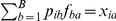, then this solution maximizes the likelihood in Equation 3. Thus, we can view the maximization of the likelihood function as an approximation of the factorization of the 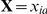 matrix, which is row-stochastic, into two other row-stochastic matrices:
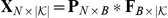
This factorization corresponds to a set of 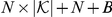 linear equations, to which each additional sample adds 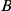 variables but also a much larger number of equations - 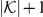; this could serve as an intuition for the advantage conveyed by sample multiplicity. In addition, this factorization is a variant of the non-negative matrix factorization (NMF) technique, with the added stochasticity constraints (so that the sum of each row in 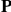 and 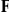 is 1). NMF is used to unveil hidden structures within data, and its major advantage over methods such as the widely-used PCA is the high interpretability of the inferred components [19]. A recent paper [20] used NMF in a metagenomic context, however the factorized data matrix was generated using alignment to sequenced genomes, in contrast to our method which does not rely on prior knowledge. The stochasticity constraints turn our model to an exact instantiation of PLSA [8], [21], a generative model from the statistical literature. PLSA was originally applied to the field of text analysis for the discovery of topics in a corpus of documents [22]. Due to its great flexibility, it was successfully applied to multiple problems in the field of text learning [23]–[25] as well as to image content analysis tasks [26].While strong similarities exist between PLSA and NMF, the fact that PLSA is based on a probabilistic model allows us to refine the model to better match the properties of the sequencing data, as we do below.

In the above procedure we extract only the first 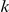-mer from each read because the model assumes that the 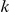-mers are sampled independently according to 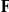, conditioned on the read's component. Extracting multiple 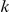-mers would result in a deviation from the model due to the dependencies between neighboring 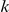-mers on the same read, as well as dependencies between 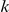-mers extracted from multiple reads covering the same genomic region.

Interestingly, the simulations presented in the Results section demonstrate that extracting multiple 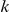-mers from the same read improves performance, despite the dependencies. The reason is that under reasonable coverage and when 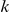 is not too large, the relative abundances 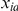 approach a constant value, and the exact sampling strategy has no effect on the final counts data. It is therefore of benefit to extract multiple 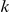-mers from each read when processing the sequencing reads, however it turns out that the best strategy is to choose not all 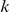-mers present on the read but only a subset, while using a slightly different model, as we explain next.

#### A refined model

Once multiple 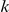-mers from the same read are used, the model can be refined to reflect the fact that all 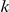-mers extracted from the same read were sampled from the same component. The likelihood function below entails this information:
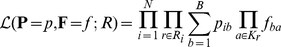
(4)where 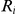 is the set of all reads sampled from sample 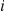, and 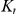 is the multiset of the 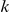-mers extracted from read 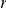.

The refined model, like the previous model, assumes that the 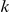-mers on a read are sampled independently given the component, but unlike the previous model, the refined model is sensitive to deviations from this assumption. The reason is that the refined model assigns each read to a component, as opposed to each 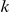-mer, and is therefore more prone to local distortions in the 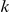-mer distribution which result from the dependencies. As we show in the Results section, an effective way to alleviate this problem is to sample a smaller number of 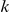-mers which are more sparsely dispersed along the read. As a result, sampling a relatively small number of 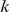-mers from each read and using the refined model turns out to be an effective strategy for using the sequencing reads in order to estimate 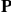.

### Parameter estimation using EM

We use Expectation-Maximization algorithms in order to approximate the maximum likelihood solutions of both the original model (defined in Equation 3) and the refined model (Equation 4), beginning with the refined. The observed variables are groups of extracted 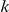-mers, one group for each read, and the latent variables are the assignments of a component to each of the reads. Let 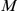 be the unknown assignments, and let 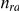 be the number of occurrences of 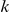-mer 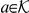 in the multiset of 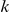-mers extracted from read 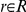, denoted 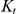. The algorithm can now be written as

**E-step:**

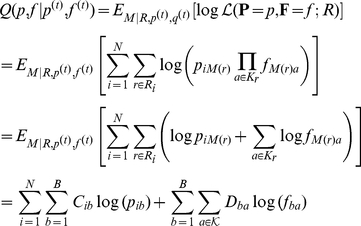
where
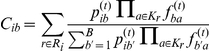


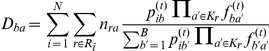



**M-step:**

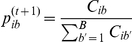


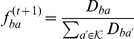



The running time of each iteration of the above algorithm is 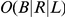, 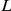 being the number of 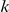-mers extracted from each read. For realistic values of 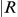 this is time consuming. In addition, for large datasets the entire data cannot fit in memory. Consider, for example, the case where the number of individuals is 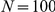, the number of reads per individual is 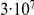, and all non-overlapping 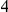-mers from reads of length 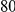 bp are used. In this case, the amount of memory required is at least 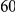 GB, even if every nucleotide letter is stored in two bits. We note that by changing the order of the summation, one can use a considerably smaller amount of memory, however in each iteration of the EM the entire dataset will have to be read again to memory. Therefore, when analyzing large datasets we recommend to use the simpler model, described by Equation 3, which ignores the relation between 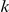-mers on the same read. The input counts 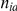 are extracted in a single pass through the data. The latent variables 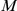 are the assignments of each pair (sample, 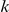-mer) to a component, and the EM algorithm is as follows:

**E-step:**

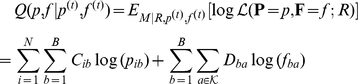
where
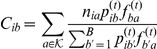


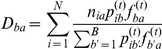



The M-step is similar to the one described for the refined model. Note that the running time of each iteration of the EM algorithm is now 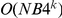, and it is therefore very efficient as long as 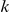 is fixed.

### Binning algorithm

The model we presented infers common components in the samples but does not assign the reads to these components; it provides for each read a probability distribution over the components that could be used for an assignment, but in general is not optimized for this goal. Such assignment, or binning, is an important preliminary step in the analysis of metagenomic samples, especially binning according to species of origin. We therefore devised an unsupervised algorithm which performs binning over multiple samples simultaneously, again leveraging on sample similarity, this time assuming a common species core. Most previous unsupervised binning methods are based on sequence composition [10]–[15]. For example, CompostBin [11] computes for each read its 6-mer distribution, similarly to the process performed by our component inference algorithm, and clusters these distributions using spectral methods. The main limitation of composition-based approaches is that they require relatively long reads (1000 bp in the case of CompostBin) due to the variance in sequence properties along the genome. Recently, a coverage-based method, AbundanceBin, was developed [16] with the advantage of being able to bin even very short reads (as small as 75 bp). Since it relies on abundance differences for binning, AbundanceBin is only able to discern between species whose abundance levels are considerably different (they report that a ratio of 2∶1 is required). Our algorithm is also coverage-based, but because it operates on multiple samples it can use abundance difference in any of the samples to tell between such species.

Assume we are given 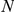 metagenomic sequencing samples, consisting of a total of 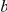 bacterial species. We wish to divide the reads in all samples into 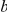 bins that correspond to the species from which they were sequenced. The binning algorithm, which we term MultiBin, proceeds as follows:

1. Pool the reads from all the samples together, and perform pairwise alignment between all pairs. For each pair, check whether the alignment shows a long overlap between the two sequence reads, suggesting that the two reads originated from the same genome. Put differently, we generate a graph 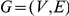, where the set 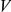 corresponds to the reads, and the set of edges 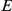 corresponds to pairs of reads with a substantial overlap. In our experiments we demanded an overlap of at least 50 bases per sequence.
2. Greedily find a maximal independent set in 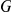. We call the reads in this set *tags* and denote it by 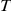. Following this process each read 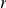 is either a tag, or is affiliated with a single tag which substantially overlaps it, 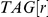. For each tag read 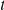, we denote by 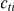 the number of reads from sample 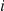 which substantially overlap it (but are not necessarily tagged by it), and by 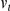 the vector 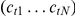.
3. Perform k-medoids clustering on the set of vectors 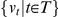, starting from a random choice of 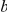 centers. Wait for convergence and divide the tags into bins according to the clustering result. Assign every non-tag read 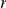 to the same bin to which 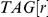 was assigned.

In the last stage, the distance between every two vectors 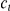, 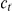 was computed as 
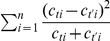
. 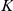-medoids clustering was performed using a local search procedure, in which we start from a random choice of centers and attempt to improve the solution by swapping at least one of the centers with another vertex, until no further improvement can be made.

The initial stage of distance computation takes 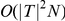, and each iteration of the clustering algorithm takes 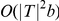; in our experiments convergence was reached within three iterations or less. The running time of the alignment stage, as well as the size of 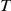, depends on the composition of the mixtures. We note that clustering is performed only on the tag reads, whose number is approximately bounded by the sum of the genome sizes in the samples divided by the read length.

The above procedure assumes that the number of species in the samples 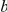 in known. In the Results section we describe a procedure for determining the number of species based on examining the clustering results for different numbers of bins.

## Results

We evaluated our methods using both real data and simulated data. We used the MetaHIT dataset (downloaded from EBI, accession ERA000116), which includes over 0.5 terabases of sequence generated from the gut microbiomes of 124 European individuals using the Illumina Genome Analyzer technology. The average amount of sequence per individual is 4.5 gigabases, and the paired-end read length is 44 or 74, depending on the sample. We used the publicly available raw reads, which were obtained after filtering human and Illumina adapter contaminant reads and low quality reads. The sampled individuals vary on the following variables: country of origin (Denmark/Spain), age, BMI (Body Mass Index), gender, and status for infectious bowl diseases (Ulcerative Colitis/Crohn's disease/disease free). In the context of this paper all the variables will be referred to as phenotypes of the human host, although country and age are in fact determinants of the metagenomic content, instead of being affected by it.

### Large differences in 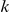-mer distributions between phenotype groups

In the initial stage we simply compared, for each 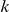-mer, its relative abundance in different phenotype groups using a two-sample *t*-test. Surprisingly, the relative frequencies of many of the 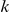-mers are significantly correlated with many of the phenotypes. This is true even for 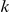 as small as 3: for example, the frequency of 69% of the 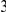-mers and of 61% of the 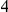-mers differs between the Spanish and the Danish samples at the 0.05 level. To the best of our knowledge, such dramatic differences in sequence composition between samples from different countries have not been observed previously. This effect might be partially due to differences in sample preparation and DNA extraction procedures, which are known to exist between the MetaHIT samples from the two countries; however, we also observed significant differences across phenotypes within each country: The frequency of 40% of the 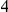-mers differs between the Crohn and the healthy Spanish samples, and the frequency of 18% of the 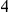-mers differs between the 10 highest- and lowest-BMI Danish groups. Permuting the phenotype labels 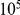 times yielded p-values of 0.0027, 0.0521 respectively for these fractions of rejected nulls.

A possible concern regarding the counts statistics are possible biases in the GC content distribution of the reads. We note that unlike different single-genome samples, different metagenomic samples are not expected to contain the same sequence composition characteristics, and therefore normalizing for such biases is a challenging task. We note that in the context of an association study between a phenotype and the metagenome, it is possible to avoid this problem using a permutation test, at the expense of power reduction.

### Multi-sample modeling of bacterial stratification

The majority of the correlations between 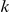-mers and phenotypes are false positives resulting from a hidden stratification which confounds the 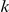-mer distributions. In order to reveal the components and to quantify them, we solved the probabilistic model described in the Methods section. Because the MetaHIT dataset is large, we used the more efficient version of the algorithm (which solves Equation 3). The input to the algorithm is a counts matrix of size [124×136], detailing for each of the 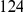 samples how many occurrences of each possible 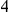-mer it includes (there are only 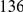 possible 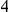-mers, instead of 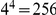, because complementary strings are indistinguishable in the sequencing data). Since extracting multiple 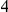-mers from each read did not seem to considerably change the results in this particular case (this does not hold in general, as illustrated later), we used only the first, highest-quality 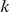-mer. The model was solved for three components (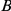 = 3) by running EM multiple times from random starting points and choosing 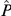 from the maximum-likelihood run. The solution 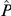 provides, for each sample 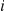, the components proportions 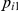, 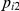 and 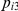, such that 
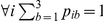
.

**Figure 1:**
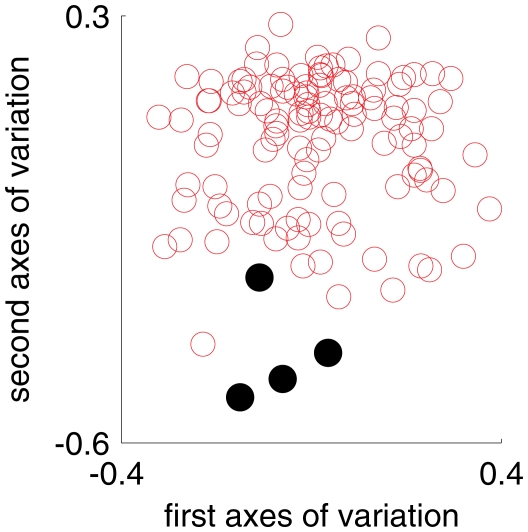
Crohn's disease status separates with components proportions. Each marker corresponds to an individual, red for Crohn-free and filled black for Crohn cases. The markers are positioned on the two-dimensional plane defined by the components proportions (there are three components but only two dimensions because the proportions sum to 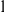). The Crohn-free individual at the bottom part of the figure is a colitis case.

We first tested each of the components 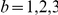 for correlation with each of the phenotypes in the following regression model:
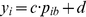
(5)We solved this model for each phenotype 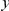 and for each cluster 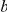, attempting to discover biologically meaningful components. Simple regression and logistic regression are used for continuous and dichotomous phenotypes, respectively. As can be seen in Table 1, highly significant p-values were obtained for predicting country among healthy individuals and for predicting BMI among the Danish. These results remain consistent also after correcting for the other measured phenotypes by entering them as covariates into the regression models. Results significant at the 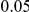 level were obtained also for predicting colitis and Crohn status among the Spanish. In the case of Crohn's disease, the power of the regression model was limited due to the small number of cases (only 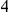), but 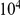 permutations yielded a p-value of 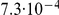. Figure 1 visually demonstrates the separation of Crohn cases and controls on the plane defined by the components proportions.

**Table 1:** Significant correlations between components proportions and phenotypes.

predicted variable	component 1	component 2	component 3
country within healthy	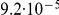 ( 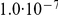 )	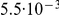 ( 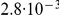 )	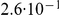 ( 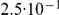 )
BMI within Denmark	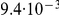 ( 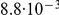 )	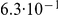 ( 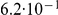 )	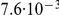 ( 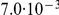 )
Colitis within Spain	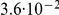 ( 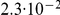 )	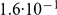 ( 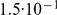 )	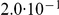 ( 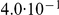 )
Crohn within Spain	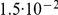 ( 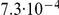 )	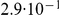 ( 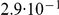 )	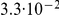 ( 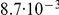 )

The predicted variables were regressed on the proportions of each component separately. The table gives the regression p-values, and in parentheses the empirical p-values obtained by permuting the components proportions 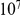 times while keeping the phenotypes constant.

We note that we also solved the model for 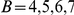. In these cases we found that the smallest per-component p-values, as well as the proportions of explained phenotypic variance captured by all components together, were similar to those obtained for 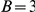. We therefore report the estimates for three components throughout the paper.

### The components estimates correct for stratification

The results from the last section, showing that the components proportions are correlated with some of the phenotypes, suggest they indeed may be used to correct the association between these phenotypes and long 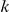-mers; we therefore attempted to perform this correction on 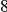-mers.

**Figure 2:**
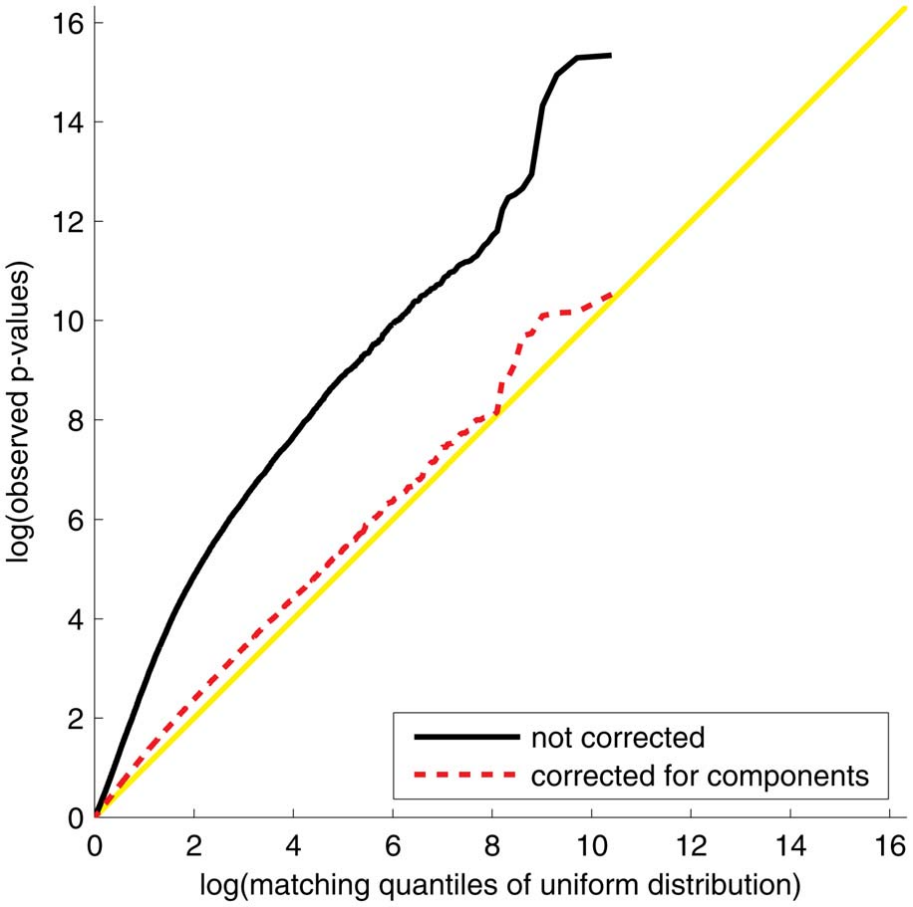
Association between 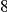
 **-mers relative abundances and BMI can be corrected using the components proportions.** Quantile-quantile curves comparing the uniform distribution to the distribution of the p-values for association between all 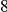-mers and BMI within the Danish samples. The uncorrected p-values are highly deflated (black), indicating that the abundance of many 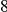-mers is correlated with BMI. However, when the components proportions are added the the regression equation (red), the correlation disappears for most 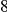-mers.

Figure 2 demonstrates such a successful correction. Two quantile-quantile curves compare the uniform distribution to the distribution of the p-values obtained by testing association between all possible 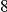-mers and BMI within the Danish samples. The black curve shows the uncorrected p-values, and its shape reflects the fact that they are highly deflated. The red curve shows the p-values obtained by adding the components proportions to the regression equation; this curve approaches the identity, indicating that these statistics capture the variance in the phenotype explained by most of the 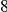-mers.

### A comparison between the likelihood models

We compared the precision of the two models, the original and the refined, utilizing different strategies for 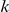-mer extraction. Performance was tested on simulated data, each simulation consisting of 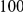 mixtures of the following 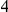 bacterial species: *Listeria monocytogenes* (phylum Firmicutes), *Bacteroides vulgatus* (phylum Bacteroidetes), *Bifidobacterium longum* (phylum Actinobacteria), *Pseudomonas stutzeri* (phylum Proteobacteria). The components distributions matrix 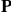 was randomly drawn from the uniform distribution and normalized to row-stochastic, and the 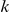-mer distributions matrix 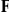 was computed from the actual genomes. For each sample 1,000 sequencing reads of length 100 bp were simulated by sampling a bacterium according to P, and then a random position in the genome of that bacterium as the starting point of the read. We chose a read length of 100 bp since it is currently a length that is obtained by most high-throughput sequencing technologies.

**Figure 3:**
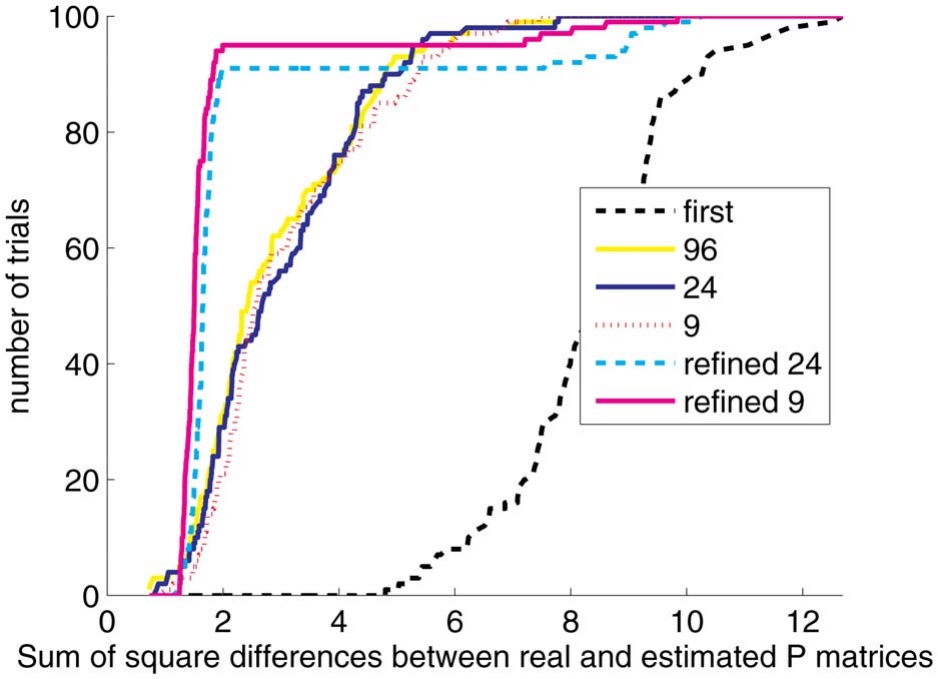
Strategies for 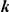
 **-mer choice under the different models.** Error is measured as the sum of squared differences between true and estimated 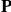 matrices. The plot presents, for different error thresholds, the number of runs out of 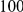 which yielded a precision at least as small as the threshold. Using the original model, we extracted from each 100 bp read either the first 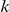-mer, 9 sparely dispersed 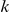-mer along it, 24 non-overlapping 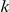-mers or 96 overlapping 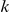-mers. Extracting multiple 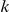-mers can be seen to increase precision considerably. Shifting to the refined model yields an even better precision; since this model is more sensitive to dependencies between 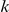-mers, extracting only the fewer dispersed 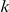-mers is preferable over extracting all non-overlapping 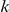-mers.

Figure 3 compares the effect of extracting different subgroups of 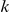-mers along each read and using them either in the original or in the refined model. Under the original model, extracting multiple 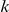-mers improves estimation precision of 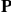 compared with extracting only the first 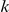-mer; this is the case even when these 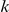-mers overlap, and are therefore highly correlated. Shifting to the refined model greatly improves the estimation precision when choosing all non-overlapping 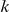-mers along the read; however, even better results are obtained when using only nine sparsely dispersed 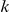-mers along the read, as using a small number of distant 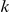-mers decreases the dependencies of 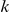-mer sequence between and within reads.

### A comparison between PLSA and PCA

PLSA is a dimensionality reduction method, and we therefore compared its performance to the widely used principal component analysis (PCA). PCA has been extensively used in metagenomic studies [2], [5] for sample visualization and classification. We used the simplified setting in which the counts matrix 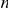 is generated from mixtures of multinomials: Setting 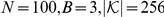 we generated 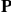 and 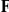 by drawing their entries from the uniform distribution followed by normalizing to row-stochastic, and then generated the counts matrix 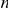 by sampling each row 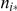 according to the multinomial distribution specified in 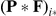, with varying numbers of counts per sample.

Both PLSA and PCA were tested in the task of estimating the matrix 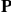. Since PCA operates with no stochasticity constraints, we estimates precision as the average squared correlation coefficient (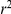) between the true vectors 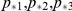 and either the three strongest principal components (for PCA) or the vectors 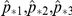 (for PLSA). For both methods we chose the ordering of the components that yielded the highest score.

**Figure 4:**
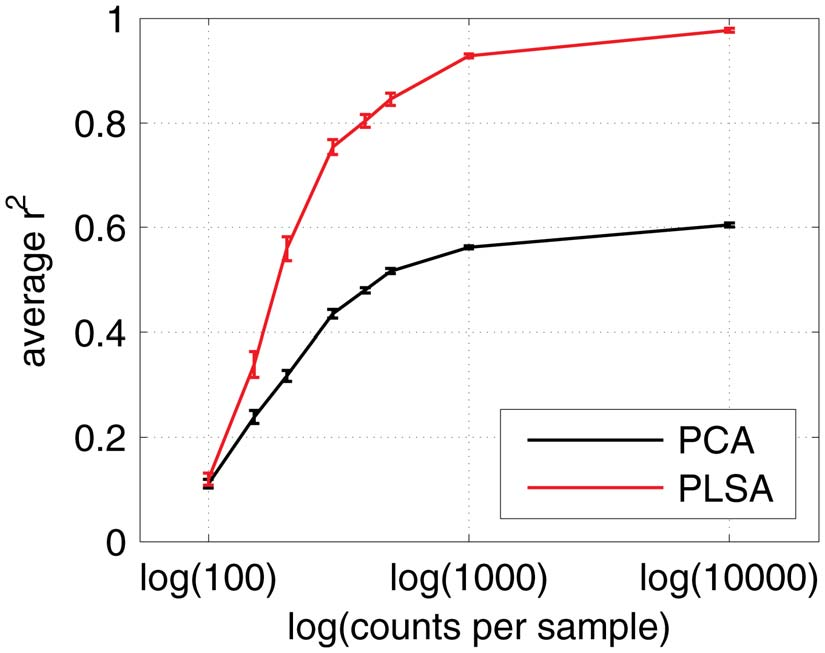
PLSA approximates mixture coefficient better than PCA. PCA and PLSA were performed on a simulated counts matrix 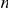 with 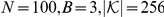 and different number of per-sample counts. The plot shows the average squared correlation coefficient between the true vectors 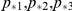 and the three strongest principal components (in the case of PCA) or PLSA estimates 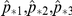. For each per-sample counts value 20 experiments were performed, and the plot gives the mean result and the standard error of the mean. The estimates obtained by PLSA show higher correlation with the true mixture proportions.

Figure 4 shows that PLSA's estimates of 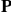 are considerably more accurate than those obtained by PCA. This result confirms that PLSA is indeed a more appropriate method for characterizing mixture components in our context.

### Joint binning over multiple samples

Nine datasets, each consisting of five metagenomic samples, were generated using MetaSim [27]. All samples in a given dataset contained the same set of bacterial species in different, randomly drawn proportions. The datasets differed in the number of species they contain, ranging from 2 species to 10 species in each sample of the most complex dataset. The species distribution of a sample containing 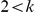 species was generated incrementally by adding a random number sampled uniformly from [0,1] to the species proportions of an existing 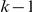 sample and normalizing to 1. In the first experiment we generated 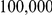 reads of length 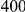 bp from each sample, and in the second experiment 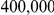 reads of length 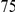 bp each. Overlaps between reads were determined by running BLAT [28] and requiring an exact match at the edges of the reads; the BLAT parameters we used restricted the results to matches of length 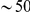 bp and above. Precision was computed as the fraction of reads assigned to the correct species, averaged over all species.

We compared the precision obtained by MultiBin to the performance of AbundanceBin, a program implementing the equivalent coverage-based approach which was shown to perform precise binning of species exhibiting different abundance levels using reads of lengths 400 and 75 bp. AbundanceBin operates on single samples only, and therefore was run separately on each sample, while MultiBin was run on all five samples in each dataset simultaneously. We note that the separate execution of AbundanceBin conveys no information about the correspondence between the bins across samples, and so we chose the best matching between the bins and the species in each sample so as to maximize the total precision.

**Figure 5:**
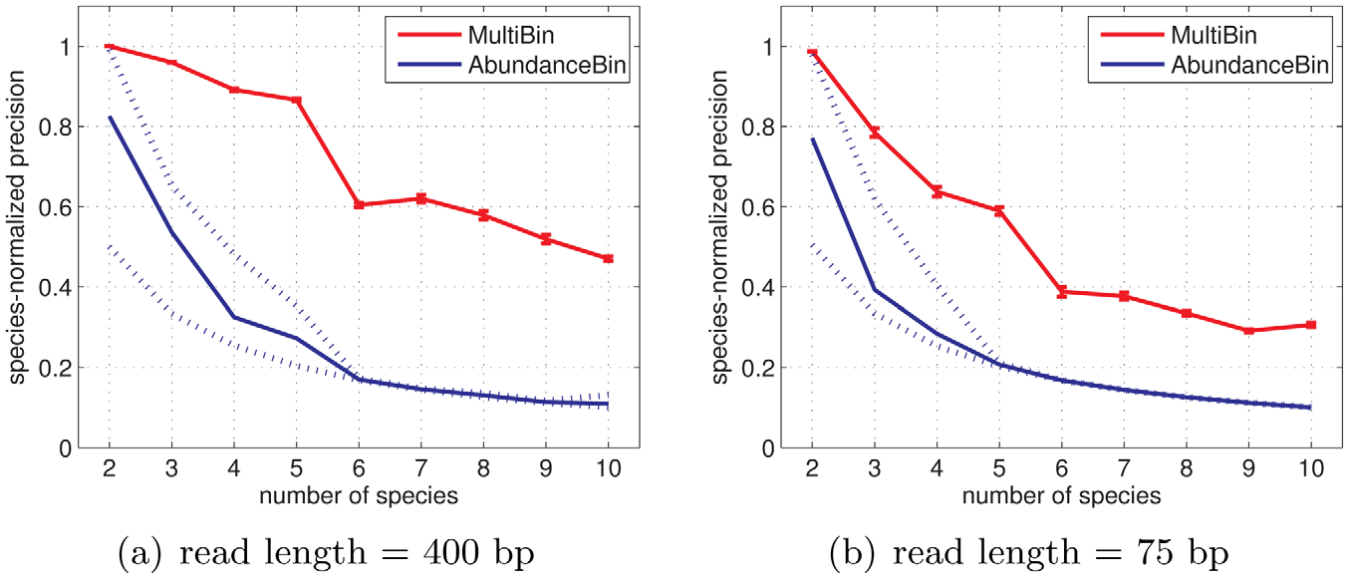
Simultaneous binning over multiple samples achieves higher precision compared with the equivalent single-sample approach. MultiBin and AbundanceBin were both run on datasets of increasing complexity. Each dataset is composed of 5 mixtures of the specified number of species. The specified precision is the proportion of reads correctly assigned to a bin, averaged over all species. For MultiBin (red) the curves show average precision over 10 random starts of the clustering algorithm, and the error bars give the standard error of the mean. For AbundanceBin (blue) the curves show the average precision over the 5 samples in the dataset, and the dashed lines give the highest and lowest result of the 5. MultiBin achieves consistently better precision over both read lengths and over all sample complexities. AbundanceBin's performance exhibits high between-sample variability, and also deteriorate more rapidly as the number of species increase.

As can be seen in Figure 5, MultiBin performs better than AbundanceBin over both read lengths and over all dataset complexities. For the 400 bp reads MultiBin maintains a precision of over 0.8 even for mixtures of five species. MultiBin is also able to bin the 75 bp reads, although with lesser success; we note that the ability to bin short reads is unique to coverage-based approaches, and that in principle there is no advantage in having longer reads, assuming the coverage is high enough. As for AbundanceBin, its performance on the simple mixtures exhibits high variation between samples because of its reliance on large abundance differences within each sample. AbundanceBin's performance also deteriorates more rapidly as the number of species per sample increases compared with MultiBin.

We went on to evaluate MultiBin under the realistic scenario in which the reads have sequencing errors. To do so, we adjusted the alignment stage of MultiBin, currently performed by BLAT, to be more permissive. We tested this modification by again generating for the above datasets 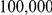 reads of length 400 bp, this time introducing base substitutions into the reads. When the substitution rate was increased to 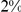 and then to 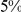, the precision for mixtures of two species decreased from 1.0000 to 0.9975 and then to 0.9966. Overall we conclude that the effect of these errors is not dramatic, and that for realistic error rates they could be largely moderated by adjusting the alignment procedure.

We note that integrating information across samples enables MultiBin to perform precise binning even when the variance in species distribution across the samples is relatively small. For example, when simulating 400 bp reads from five nearly-balanced mixtures of two species - the relative abundance of the more abundant species were 0.57, 0.55, 0.70, 0.53 and 0.56 - AbundanceBin still obtained a precision of 0.93. These results also demonstrate that MultiBin achieves precise binning on nearly-balanced samples; in contrast, a coverage-based method which did not integrate information across samples would produce extremely poor results on each of these samples alone.

Determining the number of clusters is an issue widely explored in the literature, and particularly, several approaches exist and have been tested for similar problems [29]. We found that running the algorithm multiple times for different values of 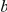, measuring the Hartigan index [29], [30] for each value and choosing the value at which the index decreases sharply and reaches a plateau gave accurate results, as long as the binning itself was accurate enough (precision of 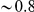 and above).

### Sample multiplicity in the design phase

Our results so far demonstrate that joint modeling of multiple metagenomic samples can be helpful in the analysis stage. A further question has to do with the design stage: Given a fixed coverage depth and a potential pool of related metagenomic samples, how many of the samples should be sequenced in order to achieve optimal characterization of the underlying microbial composition? There seems to be a tradeoff between sequencing with high coverage a small number of samples and the superficial sequencing of many samples. We tested this tradeoff in both the components estimation problem and the binning task.

**Figure 6:**
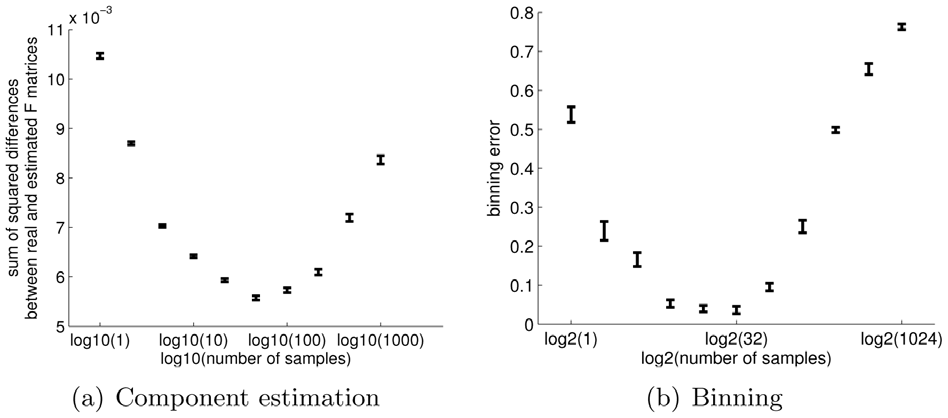
Increasing the number of samples for a fixed depth of coverage improves both components characterization and binning precision. **Left:** A fixed number of 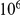 counts were generated from a model defined by uniformly drawn 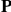 and 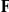 matrices using 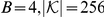. The value of 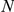, the number of samples, varied from 1 to 1000, and 100 trials were performed for each value. The highest average precision of 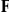 estimation is obtained for 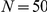. **Right:** A fixed number of 8,192,000 reads of length 400 bp were sampled from different numbers of samples, each consisting of 15 species in uniformly drawn proportions. The smallest average error over all samples was obtained when 32 samples are sequenced. In both plots the error bars give the standard error of the mean.

For components estimation, our task is to best characterize the components, or in other words to estimate the 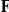 matrix; the 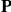 matrix varies with the number of samples and therefore cannot be compared here. We simulated instances of the proposed probabilistic model by uniformly drawing the P and F matrices followed by normalization to row-stochastic, and then drawing observations from the corresponding multinomial distributions. We used 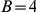 components, each defined by a multinomial distribution over 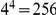 possible results. Initially, 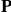 was defined for 1,000 samples, and for each sample 1,000 counts were drawn. The model was then solved for a decreasing number of samples by joining samples together to obtain 1000, 500, 200, 100, 50, 20, 10, 5, 2 and 1 samples. As can be seen in Figure 6(a), the optimal estimation of 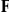 was achieved for 50 samples, each including 20,000 counts: Increasing the number of samples further past this point does not allow enough data to be gathered from each sample, resulting in a decrease in performance.

For the binning task, we ran our algorithm on mixtures of 15 species and reads of length 400 bp. Due to efficiency considerations we did not produce actual reads using MetaSim, but instead drew read start positions randomly along the genomes, and determined an overlap for reads which physically overlapped by more than 100 bp at the edges. Figure 6(b) shows the resulting precision when binning is performed over a fixed number of 8,192,000 reads allocated to an increasing number of samples, in which the species proportions are again uniformly drawn. The highest precision is obtained for 32 samples.

Both plots demonstrate that sample multiplicity is an advantage given a fixed coverage: as long as the per-sample coverage is reasonable, allocating the sequencing reads to as many samples as possible improves components characterization and binning precision.

## Discussion

We demonstrated the advantage in joint modeling of multiple metagenomic samples, by showing that it allows the unsupervised inference of hidden genetic component, and increases the precision of coverage-based binning. This advantage holds for both the analysis and the design stage; as for the latter, the results suggest that when wishing to characterize a given metagenomic sample, it is useful to divide the coverage between additional samples from similar environments. It might also be possible to apply a biological or chemical treatment to some of the samples, which would further accentuate the differences between them; when the samples are analyzed jointly, these differences are expected to further enhance performance. Similarly, sequencing data available from previous experiments can be used to improve the analysis of new samples. A similar tradeoff between the number of samples and per-sample coverage has been observed for testing the power of rare variant discovery in sequencing data [31], and sample multiplicity is likely to become a key issue in the future design of both standard and metagenomic sequencing studies.

We note that the components estimates obtained by solving the probabilistic model are interesting by themselves, outside of the context of association correction; particularly, they can allow for the characterization of variability patterns in metagenomic samples and for sample classification. Different estimates will be obtained by setting the parameters 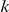 and 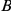 to different values; our choice of 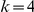 and 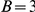 was meant to capture a high-level division, possibly taxonomic, of the microbial population, as it is known that bacterial phyla have characteristic sequence composition. A recent paper [5] identified metagenomic variability components using a supervised approach and divided the Danish samples accordingly to discrete classes termed *enterotypes*; interestingly, there are some correlations between these enterotypes and the components we obtain. For example, samples belonging to the first enterotype have a low proportion of the fourth component (p-value = 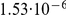) when solving the model for 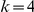 and 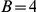, and those belonging to the second enterotype have a low proportion of the first component (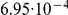) when solving the model for 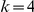 and 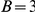. However, as explained in the Methods, our algorithm is fundamentally different from the PCA used to identify the enterotypes, and is expected to yield components of different nature, on top of it being unsupervised.

As for the proposed binning algorithm, unlike most other algorithms it can be used on datasets containing short reads, since the reads need only be long enough so as to determine unique sequence overlaps between them. In addition, the algorithm can be further improved to use not only coverage information but also other features, such as sequence composition, by adding them to the vectors on which clustering is performed.

Lastly, the implementation of the proposed association test for the MetaHIT dataset was limited by the sequencing quality, which forced us to extract only the first 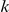-mer from each read and therefore to examine only relatively short 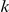-mers (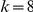), otherwise the counts data would become too sparse. We believe that this problem could be addressed by the integration of sequencing uncertainties into the counts data. With the expected improvements in high-throughput sequencing technology in terms of read length and read accuracy, these issues may be of lesser importance in the future.
